# Protective Effect of Tang Wang One Decoction on the Retinal Vessels of Diabetic Rats

**DOI:** 10.1155/2017/8635127

**Published:** 2017-03-06

**Authors:** Xinyun Kou, Shufei Yang, Yali Qin, Chao Yang, Tingting Deng, Dan Luo, Ming Jin

**Affiliations:** ^1^China-Japan Friendship Hospital, No. 2, Yinghua East Street, Chaoyang District, Beijing 100029, China; ^2^Xicheng Maternal and Child Health Hospital, No. 19, Pingyuan Street, Xicheng District, Beijing 100054, China

## Abstract

*Objective*. This study aimed to determine the influence of Tang Wang One Decoction (TWOD) on the retinal vessels of diabetic rats.* Methods*. The hemorheology of diabetic rats was observed. Morphological studies of retinal vessels were conducted using optical microscopy and electron microscopy. Immunological histochemistry assay was used to measure the expression levels of MMP-9, occludin, and claudin-5.* Results*. Obvious pathological damage was observed in the retinal vessels of diabetic rats. TWOD positively affected the hemorheology and morphology of retinal vessels. The decoction also decreased the expression of MMP-9 and increased the expression of occludin and claudin-5.* Conclusions*. The results suggest that the retinal protective effects of TWOD might be related to downregulation of MMP-9 and upregulation of occludin and claudin-5.

## 1. Introduction

Diabetic retinopathy (DR) is the most common microvascular complication of diabetes. At the early stage DR, abnormalities such as decreased red blood cell deformability and enhanced aggregation can be observed in the blood components. With the change in blood cells and vascular cell wall function, the blood retinal barrier (BRB) is damaged by the increasing vascular permeability, proliferating endothelial cells, selective apoptosis of pericytes, thickening of the basement membrane, and intercellular connections loss [1]. Essentially, the barrier of retinal vessels maintains the proper structural foundation of endothelial cells and ensures the stable number and shape of intercellular connections. The junctional protein of endothelial cells is the critical material basis for the tight junction (TJ). TJ is mainly composed of occludin and claudin. However, claudin-5 is mainly expressed in the retina. Occludin, claudin, and junctional adhesion molecules combine to form a transmembrane protein polarity complex, which regulates the substances that go in and out of the blood vessels. This complex not only provides the necessary nutrients for the layers of the retina but also removes wastes [2]. TJ injury is the main cause of BRB damage in early DR. Matrix metalloproteinases (MMPs) are a group of proteolytic enzymes which can degrade almost all extracellular matrix. Specifically, MMP-9 degrades endothelial cell tight junction proteins, playing an important role in angiogenesis [3].

Calcium dobesilate (CaD) is considered an angioprotective drug, and it has been used in the treatment of DR. CaD relieves retinal bleeding and decreases the number of capillary hemangioma, as it reduces blood viscosity and platelet activity. The drug also corrects the excessive vascular permeability of the retina, positively affecting BRB permeability. Several clinical studies have shown a decrease in the rate of progression of DR after long-term oral treatment with CaD [4]. Moreover, CaD helps in expanding horizon by reducing intraocular pressure and in improving the retina state with increasing blood flow in patients [5].

Tang Wang One Decoction (TWOD) is a widely used Chinese herbal formula in ophthalmologic clinical applications by the author's mentor. Based on a previous work, the present study is designed to observe the effects of TWOD on the retinal vessels, hemorheology, the endothelial cells and pericytes, and the expressions of MMP-9, occludin, and claudin-5 in diabetic rats.

## 2. Materials and Methods

### 2.1. Animal Model and Grouping

All procedures involving animals were conducted in accordance with the Association for Research in Vision and Ophthalmology Statement for the Use of Animals in Ophthalmic and Vision Research. Male Sprague-Dawley rats, with age of 6 weeks and weighing 200 g to 240 g, were purchased from Beijing HFK Bioscience Co. Ltd. {SCXK (Jing) 2014-0005}. After acclimation for 1 week, the rats were randomly divided into two groups: the control group with 8 rats and the diabetic group with 24 rats. After 12 hours of fasting, intraperitoneal injection of 60 mg/kg streptozotocin (STZ) (in 10 mmol/l citrate buffer, pH 4.5) was administered to the diabetic group to induce diabetes [6]. The control group received citrate buffers only. After 72 hours, the animals with blood glucose levels consistently greater than 16.7 mmol/L were considered diabetic. Diabetic rats were divided again into the model group (treated with vehicle), TWOD group (treated with TWOD), and CaD group (treated with CaD), with all groups having 8 rats. All animals had access to abundant food and water at the China-Japan Friendship Hospital Clinical Research Institute Animal Care (SYXK (Jing) 2010-0011). The body weight and blood glucose levels were recorded carefully every 2 weeks for 9 months. The general physical characteristics, including coat color, skin, bite and sup, urine, and stool, were observed and recorded as well.

### 2.2. Drugs and Reagents

TWOD consists of red ginseng,* Astragalus, Panax*, leech, earthworm,* Salvia*, and* Poria* (weight ratio 2 : 15 : 2 : 3 : 2 : 10 : 15). All ingredients of the TWOD were combined and boiled for 2 hours. After boiling, TWOD was filtered and concentrated to 1 g/ml. Then through sterilization and vacuum packaging, TWOD was stored at 4°C at the China-Japan Friendship Hospital Extracting Room.

CaD was purchased from Xi'an Lijun Pharmaceutical Co. Ltd. Anti-occludin (ab31721), anti-claudin-5 (ab53765), anti-MMP-9 (ab137867), and horseradish peroxidases (HRP, ab97069) were purchased from Abcam. The antibody diluent (s3022) was obtained from DAKO. Trypsin (No. 0458) was get from Amresco. STZ (s0130) was purchased from Sigma.

The TWOD group was treated orally with TWOD, which was diluted in 5 ml of distilled water at a daily dose of 10 g/kg and considered as a therapeutic agent for the prevention of DR. The CaD group was used as the positive control group and was treated orally with CaD at the daily dose of 250 mg/kg diluted with 5 ml of distilled water. The control group and model group were treated with 5 ml distilled water over the same treatment period. The rats receive TWOD and CaD for 9 months.

### 2.3. Hemorheology Studies

After 9 months, the rats were anesthetized with intraperitoneal injection of 10% chloral hydrate. Then, the blood samples were collected directly from the abdominal aorta of the rats for the hemorheological test, which was conducted by Dongzhimen Hospital Lab. The blood samples were tested about whole blood viscosity at three different shear rates, plasma viscosity, and erythrocytes aggregation.

### 2.4. Trypsin Digestion Method

After parts of rats were excess anesthetized, their eyes were removed for trypsin digestion. The eye samples were stored with 4% paraformaldehyde for 48 hours. After rinsing with running water for 5 minutes, the eyes were cut along the serrated edge of the ring at the back of the ciliary body, removing the anterior segment. Using the optic nerve as the center, each eye cup was divided into three parts resembling orange petals, and the retina was isolated using 0.01 mol/L phosphate buffer saline (PBS). The retina was incubated at 37°C with the digestion buffer containing 3% trypsin. After overnight of incubation, the retina was transferred to pipetted PBS and rinsed repeatedly until a layer of transparent retinal vascular network appeared. The vascular tree was set onto glass slides for microscopic observation. After air-drying, the slides were stored at 4°C for preparation.

### 2.5. Periodic Acid-Schiff Staining Studies

The retinal trypsin digests were immersed in distilled water for 3–5 minutes. First, the digests were oxidized in 1% periodic acid solution for 10 minutes and then rinsed thrice for 2 minutes with distilled water. Second, the Schiff reagent was added for 15 minutes, and the samples were washed in running water for 10–15 minutes. Third, the trypsin digests were placed in Mayer's hematoxylin for 1 minute and washed afterward. After alcohol dehydration and xylene transparent, the vascular tree was observed under a microscope.

### 2.6. Immunohistochemical Staining Method

After immersion in 0.01 mol/L PBS for 20 minutes, the retinal trypsin digests were immersed in 0.3% H_2_O_2_ for 15 minutes to block the endogenous peroxidase. The digests were washed with running water and then washed thrice with PBS for 5 minutes. The retinas were incubated overnight at 4°C with anti-occludin (1 : 100), anti-claudin-5 (1 : 1000), or anti-MMP-9 (1 : 1000) antibodies. After incubation, the retinas were washed thrice for 5 minutes with PBS. The retinas were incubated for 1 h at room temperature with HRP (1 : 1000). Then, the retinas were washed thrice for 5 minutes with PBS. Reagent A and reagent B in diaminobenzidine were mixed in the ratio of 1 : 100, and the mixture was added dropwise to the retinas. The vascular tree was observed under a microscope to ensure that it was washed properly, revealing its color. On average, washing lasted for 3–10 minutes. After alcohol dehydration and xylene transparent, the vascular tree was observed under a microscope, and images of the sample were obtained to test with mean optical density with image Pro Plus.

### 2.7. Electronic Microscopy Specimen Preparation

Parts of the rat eyes were prepared for electronic microscopic observation. The eyes were placed in 2.5% glutaraldehyde and stored at 4°C for 72 h. The samples were washed thrice for 10 minutes with PBS. Each retina was sliced (1 cm × 1 cm), and the samples were placed in 1% osmium tetroxide for 1-2 h. The samples were then washed twice for 10 minutes with double distilled water. After dehydration by alcohol and acetone, the retinas were infiltrated and embedded by epoxy resin. The slice was cut into 10 nm sample and was stained with uranyl acetate and lead citrate. The specimens were observed under a transmission electron microscope (H6010A scanning system), and images were obtained, as supported by the China-Japan Friendship Hospital Clinical Research Institute Electron Microscopy.

### 2.8. Statistical Analysis

Data were expressed as mean ± standard deviation. Results were analyzed by one-way ANOVA, followed by a LSD post hoc test for multiple comparisons. A *P* value < 0.05 was considered statistically significant.

## 3. Results

### 3.1. TWOD Does Not Affect Blood Glucose Level in STZ-Induced Rats

**Figure 1:**
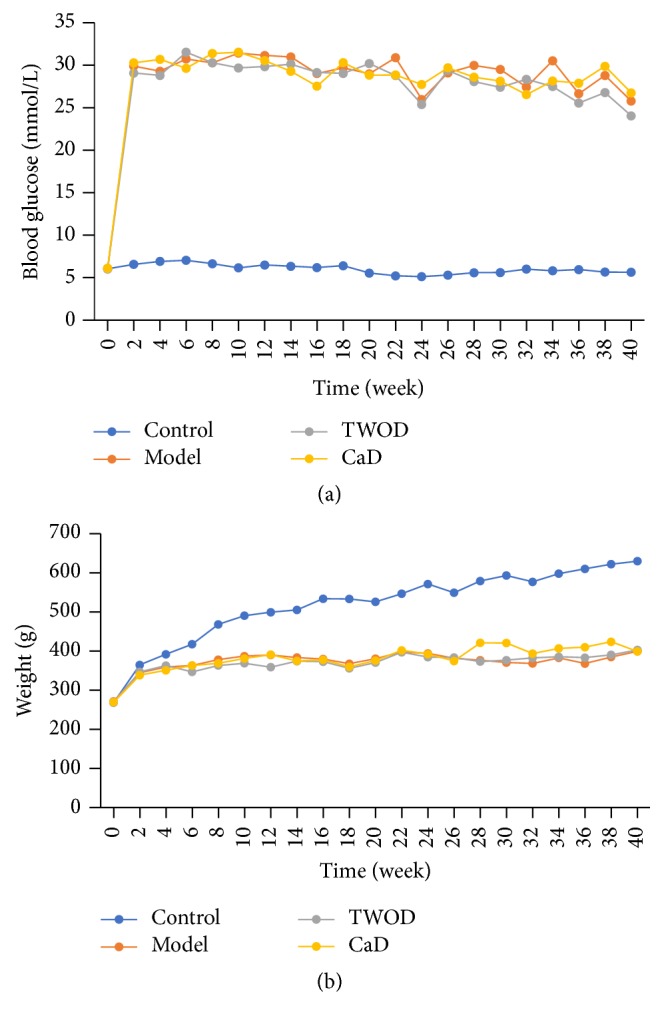
Effects of TWOD on blood glucose and weight levels in diabetic rats. Model group, TWOD group, and CaD group received a single dose of 60 mg/kg STZ to induce diabetes and control rats received vehicle. TWOD group was treated with 10 g/kg TWOD. CaD group was treated with 250 mg/kg CaD. Control group and model group were treated with the same volume of vehicle. Blood glucose (a) and weight (b) levels were recorded every 2 weeks throughout the study. Results were analyzed by one-way ANOVA, followed by a LSD post hoc test for multiple comparisons. The values are means ± SD, *n* = 8.

As shown in Figure 1, the blood glucose levels of STZ-induced diabetic rats were significantly higher when compared with the control rats, and the weight was significantly lower after six weeks. The weight and the blood glucose levels between the TWOD group and the CaD group were similar to the values obtained for the model group. The STZ-induced diabetic rat model was stable during the experimental period. The blood glucose levels of STZ-induced diabetic rats were maintained at 16.7 mmol/L or greater, and the weight was relatively stable.

### 3.2. TWOD Reduces Plasma Viscosity and Erythrocyte Aggregation in STZ-Induced Rats

**Figure 2:**
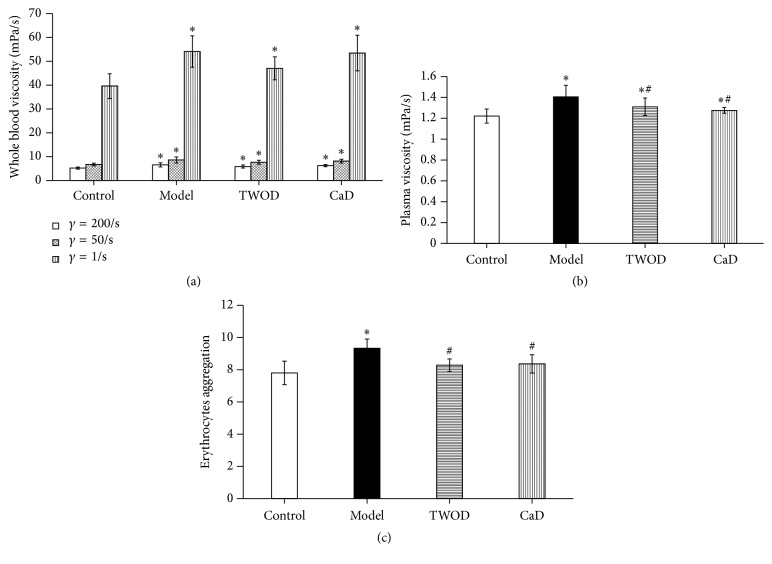
Effects of TWOD on whole blood viscosity, plasma viscosity, and erythrocyte aggregation in diabetic rats. TWOD group and CaD group received a single dose of 60 mg/kg STZ to induce diabetes and control rats received vehicle. TWOD group was treated with 10 g/kg TWOD. CaD group was treated with 250 mg/kg CaD. Control group and model group were treated with the same volume of vehicle. Whole blood viscosity (a), plasma viscosity (b), and erythrocyte aggregation (c) were tested after excessive anesthesia. Results were analyzed by one-way ANOVA, followed by a LSD post hoc test for multiple comparisons. Values are demonstrated as mean ± SD; *n* = 8; ^*∗*^*P* < 0.05 versus control group; ^#^*P* < 0.05 versus model group.

The whole blood viscosity at three different shear rates and the plasma viscosity of STZ-induced diabetic rats were significantly higher than those of the control rats. No obvious difference in the whole blood viscosity at three different shear rates was observed among the TWOD group, CaD group, and the model group. However, TWOD and CaD group significantly inhibited changes in plasma viscosity. In addition, the degree of erythrocyte aggregation in TWOD group and CaD group was significantly lower than that in the model group, as shown in Figure 2.

### 3.3. TWOD Inhibited Morphological Changes in the Trypsin Digested-Retinal Vessel of Diabetic Rats

**Figure 3:**
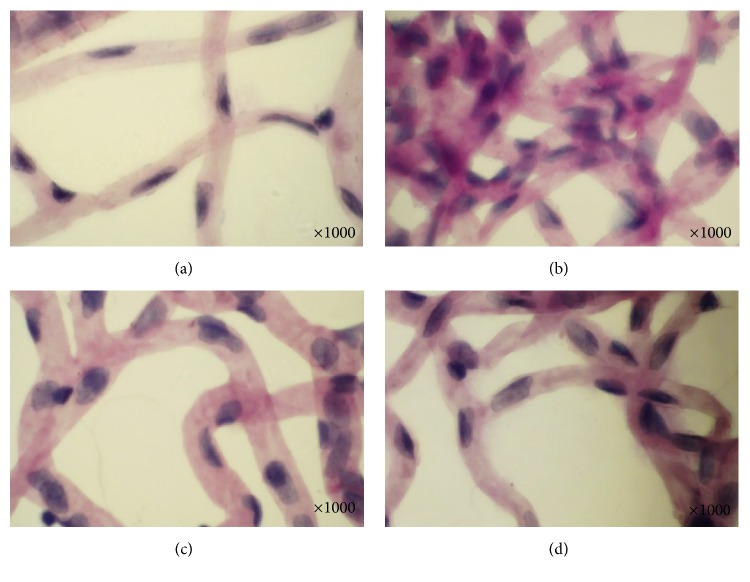
Effects of TWOD on retinal microvascular morphology in diabetic rats. TWOD group and CaD group received a single dose of 60 mg/kg STZ to induce diabetes and control rats received vehicle. TWOD group was treated with 10 g/kg TWOD. CaD group was treated with 250 mg/kg CaD. Control group and model group were treated with the same volume of vehicle. The retinas were prepared with trypsin digestion and stained with periodic acid-Schiff stain. Typical retinal microvascular staining was shown in this figure: control group (a), model group (b), TWOD group (c), and CaD group (d).

The prepared control group retinal digests showed orderly retinal capillary distribution and went straightly. The endothelial cells were generally located inside the capillaries. Under the stained light, the nuclei of the endothelial cells appeared oval with the long axis parallel to the capillary. The pericytes were generally located outside the capillary. With dark staining, the nuclei of the pericytes appeared triangular or spherical, as shown in Figure 3(a). The retinal capillaries of the model group were strikingly abnormal compared with those in the control group, as shown in Figure 3(b). Capillary networks were disorderly, with capillary hemangioma and plurality of capillary torsion gathering into cluster with vascular distortion expansion. The endothelial cells proliferated obviously, and some transformed into apoptotic bodies. Parts of the pericytes underwent apoptosis and were replaced with ghost pericytes. The TWOD group showed relatively ordered capillary arrangement compared with the model group. The decrease in pericytes and endothelial cell proliferation was not obvious. Ghost pericytes, capillary microaneurysms, endothelial cell, and pericyte apoptosis were not observed in TWOD group, as shown in Figure 3(c). The morphometry of retinal capillaries in the CaD group was similar to that of the TWOD group, as shown in Figure 3(d).

**Figure 4:**
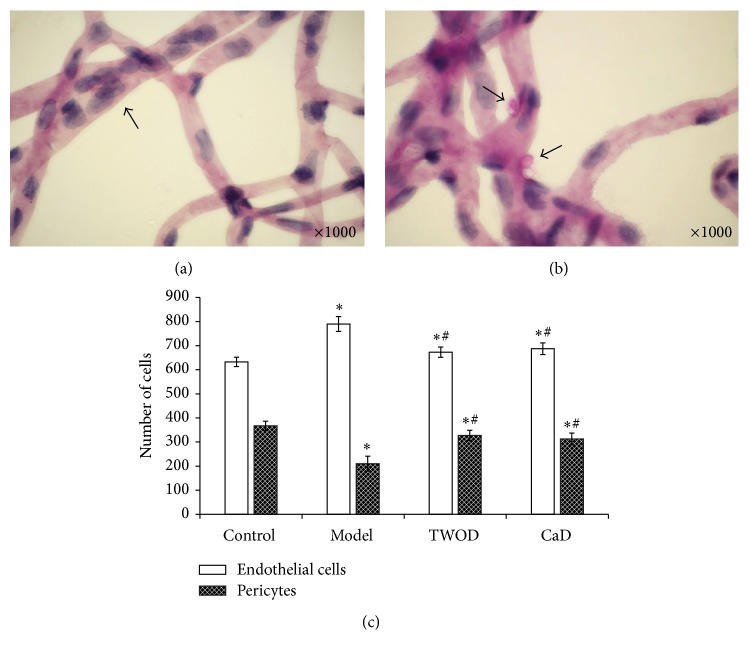
Effects of TWOD on the number of endothelial cells and pericytes. TWOD group and CaD group received a single dose of 60 mg/kg STZ to induce diabetes and control rats received vehicle. TWOD group was treated with 10 g/kg TWOD. CaD group was treated with 250 mg/kg CaD. Control group and model group were treated with the same volume of vehicle. The retinas were prepared with trypsin digestion and stained with periodic acid-Schiff stain. It was observed that endothelial cells proliferated (a). Parts of the pericytes underwent apoptosis and were replaced with ghost pericytes (b). Endothelial cells and pericytes were counted under a microscope (c). Results were analyzed by one-way ANOVA, followed by a LSD post hoc test for multiple comparisons. Values are demonstrated as mean ± SD; *n* = 8; ^*∗*^*P* < 0.05 versus control group; ^#^*P* < 0.05 versus model group.

Under the optical microscope and at 400x, the endothelial cells and pericytes along the randomly collected retina aorta stem were counted, counting 1000 capillary cells from the center to the outer periphery, as shown in Figure 4. Quantitative analysis demonstrated that the number of endothelial cells in model group (789.67 ± 36.45) significantly increased compared with that of the control group (632.67 ± 22.72). The number of pericytes in the model group (210.33 ± 36.45) was significantly lower than that in the control group (367.33 ± 22.72). The TWOD and CaD inhibited the significant reduction in endothelial cell proliferation and relative increment of pericytes compared with the model group. The numbers of endothelial cells and pericytes in the TWOD group were near the values obtained for the CaD group.

### 3.4. TWOD Alleviates the Ultrastructural Changes in Diabetic Rats

**Figure 5:**
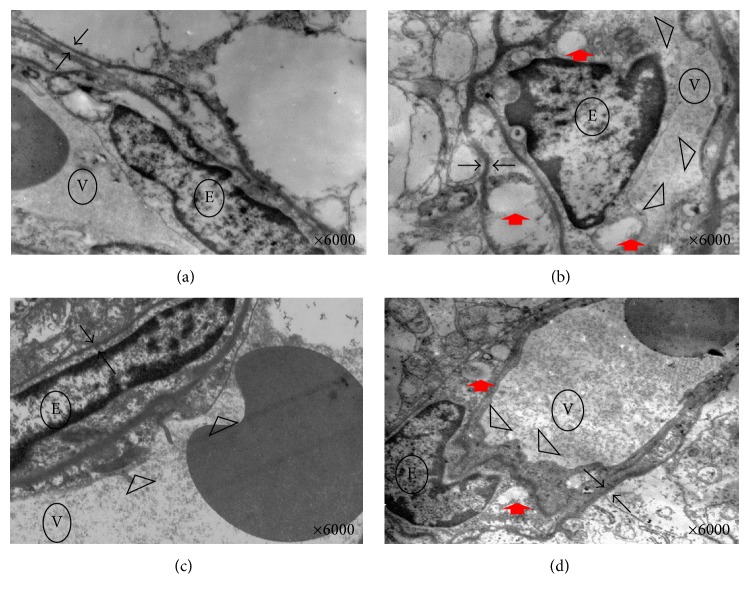
Effects of TWOD on ultrastructural changes in diabetic rats. TWOD group and CaD group received a single dose of 60 mg/kg STZ to induce diabetes and control rats received vehicle. TWOD group was treated with 10 g/kg TWOD. CaD group was treated with 250 mg/kg CaD. Control group and model group were treated with the same volume of vehicle. The retinas were prepared for electronic microscopic observation. Typical retinal ultrastructure was shown in this figure: control group (a), model group (b), TWOD group (c), and CaD group (d). Two black arrows correspond to basement membrane. Red arrows correspond to vacuoles. Triangles correspond to microvilli-shaped protuberance. ○E: endothelial cells; ○V: vessels.

The retinas were photographed and enlarged to 6000x magnification for further examination. The electronic microscopy specimen in the control group showed the presence of thin endothelial cell cytoplasm and pinocytotic vesicles and organelles. Outside the endothelial cell, pericytes appeared fusiform, and organelles were observed in the cytoplasm of the pericytes. The basement membrane is coated with endothelial cells and pericytes. In some instances, red blood cells and platelets could be seen in the vessel, as shown in Figure 5(a).

The endothelial cells in the model group were swelled with microvilli-shaped protuberance, narrowing the vessel. A large vacuole appeared in the cytoplasm of the endothelial cells. The pericytes swelled as well. In other cases, such organelles disappeared. The basement membrane was thick, as shown in Figure 5(b).

After nine months of treatment, lesions in the diabetic rats with TWOD were obviously alleviated. The endothelial cells and pericytes displayed minor swelling but without the appearance of a vacuole. The organelles were normal. Minimal microvilli-shaped protuberance was observed at the cell wall. The basement membrane thickened a little, as shown in Figure 5(c).

The ultrastructures in the CaD group were similar to those in the TWOD group except for the presence of vacuoles in the CaD group, as shown in Figure 5(d).

### 3.5. TWOD Decreases MMP-9 and Increases Occludin and Claudin-5 in the Retina of Diabetic Rats

**Figure 6:**
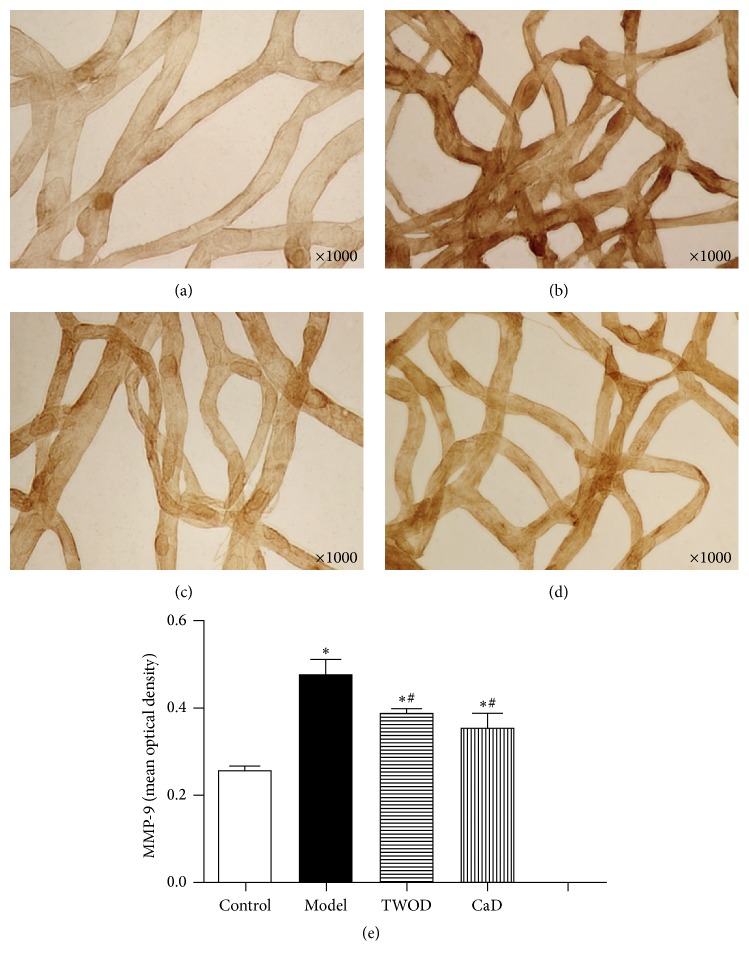
Effects of TWOD on MMP-9 levels in diabetic rats. TWOD group and CaD group received a single dose of 60 mg/kg STZ to induce diabetes and control rats received vehicle. TWOD group was treated with 10 g/kg TWOD. CaD group was treated with 250 mg/kg CaD. Control group and model group were treated with the same volume of vehicle. The retinas were prepared with trypsin digestion and stained with immunohistochemistry stain. The expression of MMP-9 was shown in this figure: control group (a), model group (b), TWOD group (c), and CaD group (d). Results were analyzed by one-way ANOVA, followed by a LSD post hoc test for multiple comparisons. Values are demonstrated as mean ± SD; *n* = 8; ^*∗*^*P* < 0.05 versus control group; ^#^*P* < 0.05 versus model group (e).

Immunohistochemistry studies showed that MMP-9 was widely expressed in the retinal capillaries of rats, as shown in Figure 6. Brown positive stain was showing through the capillary walls of the retina. Retinal expression of MMP-9 in diabetic rats receiving treatments with TWOD and CaD was significantly higher than that in the control group but significantly lower than the levels in model group. Effects of treatment with TWOD and CaD showed no significant difference.

Occludin and claudin-5 were expressed in the retinal capillaries of rats, demonstrating patchy distributions. Positive staining resulted in the brown color of the samples. The retinal expressions of occludin and claudin-5 significantly decreased in the diabetic rats compared with those in the control group. However, TWOD and CaD treatment significantly attenuated the alterations in occludin and claudin-5 expressions induced by diabetes. The expressions of occludin and claudin-5 in the TWOD group were higher than those in the CaD group.

## 4. Discussion

This study was designed to investigate the effects of TWOD on the retinal vessels of diabetic rats. Although TWOD did not decrease the blood glucose levels and weight of diabetic rats, it inhibited the changes in hemorheology, endothelial cells, and pericytes and reduced the expression of MMP-9 in diabetic rats. TWOD also positively affected the endothelial cells, pericytes, and TJ, maintaining the permeability of the BRB and delaying the development of DR.

Diabetes in rats was induced with the intraperitoneal injection of STZ, which selectively damaged the pancreatic beta cells, leading to cell death caused by the decrease in insulin and increase in blood glucose levels. The weight and blood glucose levels of the diabetic rats were stable during the nine-month experiment. No difference was observed among the blood glucose levels of the model group, TWOD group, and CaD group, and their blood glucose were constantly over 16.7 mmol/L. However, sustained high blood glucose enhances the glycated hemoglobin level in red blood cells, which may decrease the ability of red blood cells in carrying oxygen. At the same time, the increase in glycated hemoglobin changes the structure and decreases the fluidity of red blood cells, increasing blood viscosity. These changes increase the shear stress on the endothelial cells, eventually damaging the vascular wall. The injuries on the endothelial cells and pericytes caused by hyperglycemia promote vascular occlusion and avascular zone formation, enhancing tissue hypoxia. With the increase in perfusion by vascular compensatory expansion, retinal veins become dilated by distortion and appear beaded-like [7]. We discovered that hemorheology of diabetic rats was obviously changed, but TWOD prevented the changes in plasma viscosity and erythrocyte aggregation. The protective effects of the treatment are due to the presence of earthworm active protein, which improved the function of hemorheology in reducing blood viscosity, hematocrit, sedimentation equation constants, and erythrocyte aggregation [8]. Evidence showed that* Panax notoginseng* saponins and ginseng saponin could decrease whole blood viscosity, plasma viscosity, and erythrocyte sedimentation rate and relieve hypercoagulability, which inhibit thrombosis and improve hemorheology [9, 10].

In this study, we confirmed the typical pathological changes, including endothelial cell injury, pericyte apoptosis, capillary hemangioma, and vascular caliber tortuousness, in the DR of diabetic rats via retinal digests stained by periodic acid-Schiff. Hemorheological changes damaged the vessel cell walls. Endothelial cell injury is the principal pathological change characterizing DR, eventually leading to the development of vascular occlusion and avascular zone, as the disease progresses. Vascular leakage, capillary hemangioma, small bleeding points, and cotton wool spots are related to early pericyte apoptosis. Endothelial cells and pericytes are interrelated and interdependent. With the development of diabetes, endothelial cell apoptosis, avascular zone, and retinal ischemia and hypoxia occur, inducing vascular endothelial growth factor synthesis and release, which promote the proliferation and migration of vascular endothelial cells that eventually form new blood vessels [11, 12]. We observed that, with the treatment of TWOD, retinal capillaries of diabetic rats followed an orderly distribution. Excessive vasodilation, endothelial cell proliferation, and pericyte apoptosis were not obvious, given that* Astragalus* polysaccharide could protect vascular endothelial cells, which may be related to the AMPK-e NOS signaling pathway [13]. Previous studies also showed that* Astragalus* flavonoids significantly inhibited DNA injury of bovine retinal pericytes cultured in high glucose [14].

We observed swelling of endothelial cells, microvilli-shaped protuberance, degeneration of pericytes, cytoplasmic vacuoles, thick basement membrane, vascular stenosis, and vascular occlusion in the retinas of the model group under electronic microscopy. A recent study has shown that the ultrastructures, particularly the endothelial cells and pericytes, of diabetic rats changed after 12 weeks, showing chromatin condensation and margination accompanied by thickening of the basement membrane. Furthermore, the changes observed after 24 weeks were similar to those in this study [15]. Extensive research has verified that these changes in the endothelial cells and pericytes increased vascular permeability. Wrapping the endothelial cells from the outside, the basement membrane regulates the materials going in and out of the cells and sustains vascular wall permeability. Hyperglycemia damaged and thickened the basement membrane, resulting in exudative retinal disease [16]. We observed that the endothelial cells and pericytes in the TWOD group swelled a bit but with normal organelles in the cytoplasm. The thickening of the basement membrane and cytoplasmic vacuoles was not obvious. Thus, TWOD plays a positive role in protecting the endothelial cells and pericytes of diabetic rats.

MMPs, which function in the degradation of extracellular matrix components, are a group of zinc-dependent peptide chain endonuclease and proteolytic enzymes involved in wound healing, tissue remodeling, angiogenesis, scar formation, and a variety of physiological and pathological cell processes. A recent study supported the notion that ischemia and hypoxia induce the accumulation of inflammatory cells in the retinal tissue and excessive expression of cytokines, such as neutrophils and macrophages, which might promote MMP-9 release [3]. With the degradation of the vascular matrix, MMP-9 increases vascular wall permeability, leading to retinal tissue edema and inflammatory cell and endothelial cell migration, proliferation, and synthesis of more MMP-9, which favor the formation of neovascularization [17]. Our data clearly suggest that the expression of MMP-9 in the model group significantly increased, and TWOD suppressed the excessive expression of MMP-9. Occluding and claudin expressions at the transcriptional level, the protein levels, and intracellular distribution were subject to the regulation of MMPs [18]. After induction by hyperglycemia, MMP-9 degraded the extracellular matrix, collagen IV, basement membrane, and TJ protein and enhanced BRB permeability [19, 20]. In turn, TJ injury and basement membrane thickening promoted endothelial cell proliferation and migration and induced angiogenesis. Thus, diabetes damaged the TJ protein, and occludin and claudin-5 injuries accelerated the development of diabetes. In addition, several studies suggested that earthworm*, Poria,* and leech could inhibit the excessive expression of MMP-9 [21, 22].

**Figure 7:**
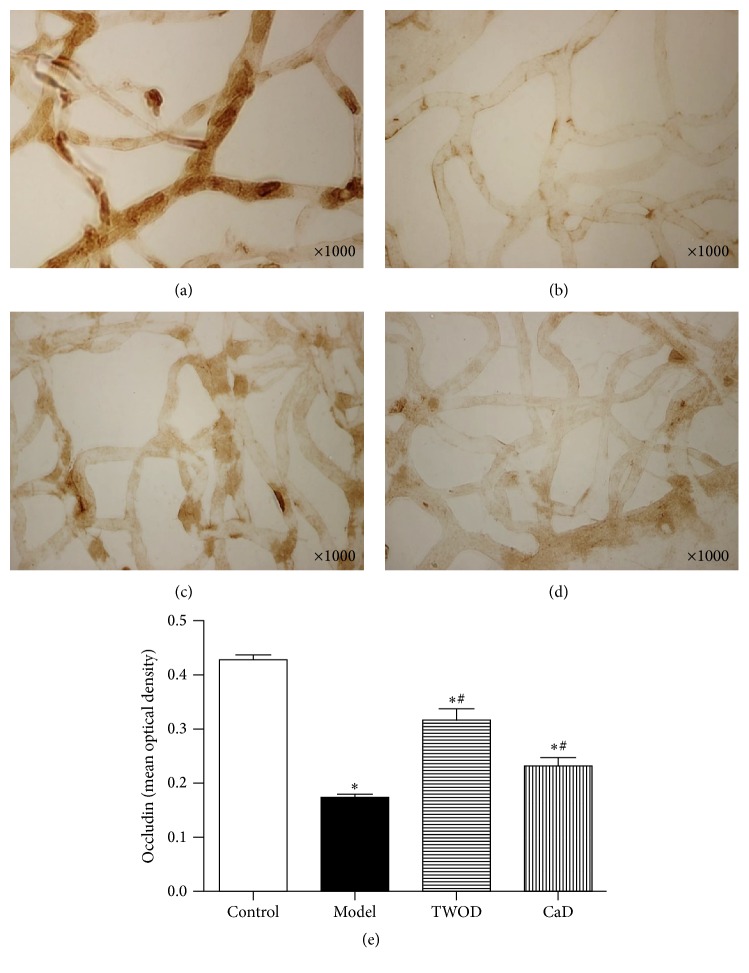
Effects of TWOD on occludin levels in diabetic rats. TWOD group and CaD group received a single dose of 60 mg/kg STZ to induce diabetes and control rats received vehicle. TWOD group was treated with 10 g/kg TWOD. CaD group was treated with 250 mg/kg CaD. Control group and model group were treated with the same volume of vehicle. The retinas were prepared with trypsin digestion and stained with immunohistochemistry stain. The expression of occludin was shown in this figure: control group (a), model group (b), TWOD group (c), and CaD group (d). Results were analyzed by one-way ANOVA, followed by a LSD post hoc test for multiple comparisons. Values are demonstrated as mean ± SD; *n* = 8; ^*∗*^*P* < 0.05 versus control group; ^#^*P* < 0.05 versus model group (e).

**Figure 8:**
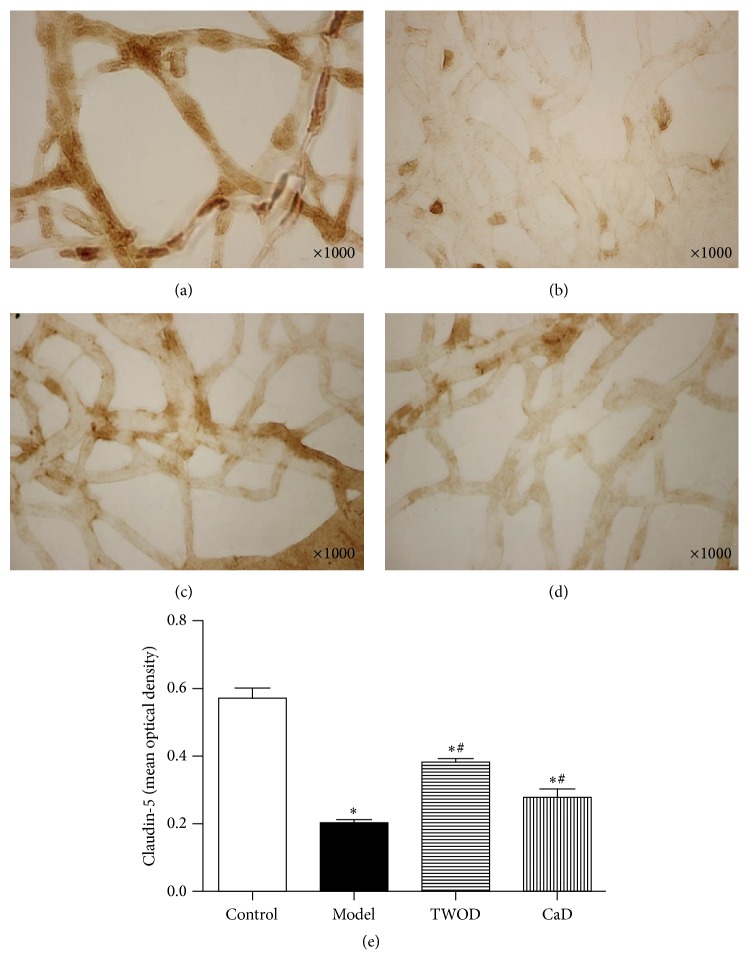
Effects of TWOD on claudin-5 levels in diabetic rats. TWOD group and CaD group received a single dose of 60 mg/kg STZ to induce diabetes and control rats received vehicle. TWOD group was treated with 10 g/kg TWOD. CaD group was treated with 250 mg/kg CaD. Control group and model group were treated with the same volume of vehicle. The retinas were prepared with trypsin digestion and stained with immunohistochemistry stain. The expression of claudin-5 was shown in this figure: control group (a), model group (b), TWOD group (c), and CaD group (d). Results were analyzed by one-way ANOVA, followed by a LSD post hoc test for multiple comparisons. Values are demonstrated as mean ± SD; *n* = 8; ^*∗*^*P* < 0.05 versus control group; ^#^*P* < 0.05 versus model group (e).

Retinal vascular damage caused by hyperglycemia was observed not only in the endothelial cells and pericytes but also in TJ proteins. In the current work, we observed that diabetes decreased retinal occludin and claudin-5 levels in nine-month-old rats. Occludin and claudin, which are found early in TJ proteins, are crucial determinants of the TJ permeability properties of endothelial cells. TJ is responsible for the direct cell-to-cell attachment. The protein maintains the concentration gradient on both sides of the cell and cell polarity and determines the permeability of cell bypass passage and ion-selection. Through signaling mode, TJ regulates the shape and size of cells and cell proliferation [23]. We discovered that TWOD prevented the decreased expression of occludin and claudin-5 in diabetic rats (Figures 7 and 8). Evidence has shown that salvianolate protects barrier permeability, which was correlated with the decreased MMP-9 levels and increased occludin and claudin-5 levels through increasing the body's antioxidant capacity and reducing tissue edema and nerve injury [24].

## 5. Conclusion

In this study, we discovered that TWOD could have protective effects on retinal vessels of diabetic rats, inhibiting changes in the morphology and quantities of endothelial cells and pericytes and abnormal hemorheology. The possible mechanisms of TWOD include decreasing MMP-9 expression and increasing occludin and claudin-5 expression. TWOD inhibited the destruction of vascular permeability by diabetes, reduced the pathological manifestations of DR, and relieved the development of DR. Results may be attributed to the effect of TWOD in tonifying qi, invigorating blood circulation, stimulating yang, and relaxing the veins. However, there are lots of questions that need to be answered before we elucidate the mechanisms of TWOD protecting the diabetic retina.
